# Cognitive impairments and their impact on daily life in persons with severe mental illness in long-term care: an explorative study using electronic clinical files

**DOI:** 10.1192/j.eurpsy.2025.2189

**Published:** 2025-08-26

**Authors:** L. Sanders, T. Van Brouwershaven, S. Piening, L. Van der Meer

**Affiliations:** 1 Department of Rehabilitation, Lentis Psychiatric Institute, Zuidlaren; 2 Department of Youth Mental Health and Autism Jonx, Autism Team Northern- Netherlands, Lentis Psychiatric Institute; 3 Department of Clinical and Developmental Neuropsychology, University of Groningen, Groningen, Netherlands

## Abstract

**Introduction:**

In persons with severe mental illness (SMI) in long-term care, cognitive impairments may be severe and pervasive. However, it remains unclear how cognitive impairments specifically impact daily life and how these effects are reflected in their everyday functioning. Collection of such data is complicated by the fact that these individuals are often excluded from scientific research and tend to drop out more frequently due to the severity and complexity of their mental health issues. This calls for a different approach, one that makes greater use of the available structured and unstructured data from electronic patient records (EPRs).

**Objectives:**

The goals of this research are to assess how cognitive impairments and their impact on daily life in persons with SMI are qualitatively addressed in EPRs and to assess whether and how such data may be systematically evaluated.

**Methods:**

We will conduct an explorative, retrospective EPR study focused on persons with SMI who use long-term care services within Lentis Psychiatric Institute, Department of Rehabilitation. EPRs contain qualitative (such as written reports) as well as quantitative (such as Routine Outcome Measures) data. To ensure patient privacy, the data in the obtained files will be de-identified. Indicators of cognitive impairments and their impact on daily life will be operationalized in collaboration with health care professionals and clustered in the domains of working memory, attention, verbal learning and memory, reasoning and problem solving, processing speed and social cognition. Using these operationalizations, natural language processing, an innovative machine-learning technique used to understand and interpret human language, will be used to identify patterns (such as, potentially, gender differences) with respect to cognitive impairments and their impact on daily life functioning from the qualitative EPR data.

**Results:**

As the study is work in progress, preliminary results are currently not yet available and will be presented at the conference.

**Conclusions:**

EPRs are a potentially vital but underused source of data for persons with SMI in long-term care. By analysing data from EPRs we may gain a broader insight into cognitive impairments and their impact on daily life in persons with SMI. Such insights are essential to aid recovery for persons with SMI in long-term care.

**Disclosure of Interest:**

None Declared

